# Prevalence and Risk Factors of Total Parenteral Nutrition Induced Hyperglycemia at a Single Institution: Retrospective Study

**DOI:** 10.1089/met.2019.0040

**Published:** 2020-05-28

**Authors:** Muna Alchaer, Rawia Khasawneh, Rochelle Heuberger, Susan Hewlings

**Affiliations:** Nutrition and Dietetics Program, School of Rehabilitation and Medical Sciences, The Herbert H. & Grace A. Dow College of Health Professions, Central Michigan University, Mount Pleasant, Michigan, USA.

**Keywords:** total parenteral nutrition, hyperglycemia, BMI, risk factors, elevated blood glucose

## Abstract

***Background:*** Total parenteral nutrition (TPN) provides full nutrition support to critically ill patients with an impaired digestive tract. Patients who receive TPN support are at higher risk for complications such as hyperglycemia. In our study, we aim to assess the prevalence of hyperglycemia induced by TPN and identify its risk factors in hospitalized adult patients.

***Methods:*** Patients who received TPN between January 2012 and December 2017 at University of Pittsburgh Medical Center—St. Margaret hospital were retrospectively screened. TPN-induced hyperglycemia was confirmed whether blood glucose was ≥180 mg/dL at any point, from the time of TPN initiation until 1-day post TPN termination. Characteristics of the hyperglycemia and the nonhyperglycemia groups were analyzed to predict potential risk factors.

***Results:*** A total of 197 patients were screened, 55 were excluded (1 died, 37 diabetic, and 17 had elevated blood glucose before TPN), and 142 patients were included, 42 of them (29.6%) developed hyperglycemia following TPN administration. Duration of TPN, surgical indications, and obesity were significantly higher in the hyperglycemia group. Additionally, age and steroids use were independent predictors of hyperglycemia in TPN patients after applying multivariable logistic regression model on our sample.

***Conclusions:*** Hyperglycemia is common after TPN. Risk factors assessment may help optimizing glycemic control in higher risk individuals to improve their outcomes. These include patients with obesity, surgical indication of TPN, and longer duration of TPN.

## Introduction

Total Parenteral Nutrition (TPN) is an essential lifesaving tool that infuses dextrose, amino acids, lipids, electrolytes, vitamins, and minerals intravenously. It provides full nutrition support to critically ill patients with an impaired digestive tract incapable of tolerating or absorbing an oral diet.^1–4^

Despite its significant benefits, TPN is associated with multiple metabolic complications.^3^ Hyperglycemia, defined as blood glucose value greater than 180 mg/dL or 10 mM per the American Society for Parenteral and Enteral Nutrition (A.S.P.E.N.) guidelines, is one of the metabolic complications that is common among adult patients receiving TPN treatment^5,6^ with an approximate incidence rate of 17%–67%.^7–12^

Hyperglycemia and its metabolic consequences can lead to adverse outcomes. A systemic literature review conducted by Capes et al.^13,14^ demonstrated that elevated blood glucose is linked to higher risk of in-hospital mortality rates after myocardial infarction and stroke incidents. Moreover, elevated glucose concentrations were shown to be associated with impairment to the body's immune response leading to higher risks of infection.^3,15^ Multiple studies have shown that TPN-induced hyperglycemia is associated with various morbidities such as cardiac complications, pneumonia, acute renal failure, respiratory failure, systemic sepsis, and death.^7,10,16–18^

Multiple mechanisms for the elevation of blood glucose with TPN have been suggested. Patients who require TPN can become critically ill, malnourished, and are often given TPN preparations with a high glucose content. This can lead to a stressed state that is characterized by an increase in the concentration of counterregulatory hormones and cytokines, causing elevated serum glucose through decreased glucose uptake in the skeletal muscles with an increase in the hepatic glucose production.^16,19–21^

The aim of this study is to assess the prevalence of hyperglycemia induced by TPN in hospitalized nondiabetic adult patients. In addition, we would like to identify the risk factors that make people more susceptible to develop this side effect.

## Materials and Methods

### Study design

We conducted a retrospective study at University of Pittsburgh Medical Center (UPMC)—St. Margaret Hospital during a 6-year period from January 2012 through December 2017. The Institutional Review Board (IRB) of UPMC granted the permission for our study.

### Data collection and analysis

We retrospectively reviewed electronic medical records of adult patients who received TPN during the study time frame. We excluded patients who had diabetes mellitus listed in their medical history. Also, we excluded the nondiabetic patients who already had hyperglycemia before receiving TPN, which was defined as the presence of a single blood glucose value greater than 180 mg/dL during the 3-day period before TPN initiation.

We considered patients to have TPN-induced hyperglycemia if they have a blood glucose value higher than 180 mg/dL at any point in time, starting from TPN initiation until 1-day post TPN termination.

The patients who developed hyperglycemia after TPN were categorized as hyperglycemia group while those who did not have elevated blood glucose post TPN were categorized as nonhyperglycemia group.

We studied the features of each group to identify the potential risk factors that might have led to hyperglycemia. We compared patients' demographics, TPN indications, TPN duration, and TPN components between the two groups. We also conducted a comparison in body mass index (BMI) based on the BMI classifications: underweight (BMI <18.5), normal weight (BMI 18.5–24.9), overweight (BMI 25.0–29.9), and obesity (BMI ≥30). Additionally, we looked at other potential contributing factors such as concurrent steroids use, history of previous TPN exposure in the past 2 years before admission, and whether the patient was admitted to an intensive care unit (ICU) or to a regular medical floor during hospitalization.

### Statistical analysis

We employed Statistical Package for the Social Sciences (SPSS) version 25 (IBM, Armonk, NY) for our study's statistical analyses. We applied two-sample *t* test, two-sample proportional *z* test, and chi-square tests to explore the statistical differences in the variables between patients who had TPN-induced hyperglycemia and those who did not. Chi-square test was used to identify the difference between categorical variables. Two-sample *t*-test was also applied on continuous variables. Two-sample proportional *z* test was used to test the proportions' difference for subcategories in BMI. Moreover, we conducted multivariable logistic regression model on independent variables thought to be potential risk factors for developing hyperglycemia. The statistical significance was defined when *P*-value was <0.05.

## Results

**FIG. 1.**
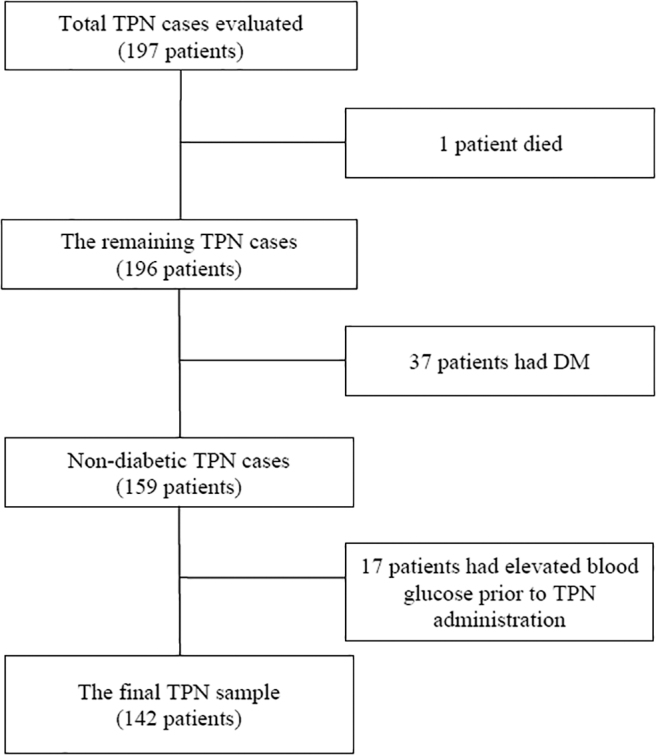
Study flowchart. A total of 197 TPN patients were evaluated. Of these, 55 patients (1 patient died, 37 patients with DM and 17 patients with elevated blood glucose before TPN initiation) were excluded. Our final sample included 142 patients. DM, diabetes mellitus; TPN, total parenteral nutrition.

We evaluated 197 patients who received TPN treatment between January 2012 and December 2017. We excluded one patient who died 1 day after TPN administration. Out of 196 patients, we excluded 37 patients who had diabetes mellitus reported in their past medical history. Out of the remaining 159 patients, we identified and excluded 17 cases that had an elevated blood glucose value greater than 180 mg/dL during the 3-day period before TPN administration. The final sample included 142 patients who were nondiabetic and did not have hyperglycemia before TPN initiation. A study flowchart is demonstrated in Fig. 1.

### General characteristics

The patients' ages ranged between 26 and 98 years with a mean ± standard deviation (SD) age of 69.5 ± 14 years. The sample had 82 women (58%) with white race predominance (131 patients, 92%).

The BMI of our sample had a mean ± SD of 27.5 ± 8.4 kg/m^2^. The patients' BMI were distributed as the following: 13 (9.2%) underweight, 45 (31.7%) normal weight, 32 (22.5%) overweight, and 52 (36.6%) obese.

The reasons for TPN initiation were divided into two groups: medical and surgical (Table 1). The gastrointestinal surgeries formed the majority of TPN indications (83 patients, 58.5%), while the rest of the indications were medical reasons (59 patients, 41.5%).

**Table 1.** Indications of Total Parenteral Nutrition Therapy in the Sample

TPN indications	n (% out of 142 patients)
Surgical	
Gastrointestinal surgeries	83 (58.5)
Medical	
Small bowel obstruction	27 (19)
Feeding tube malfunction	7 (5)
Malnutrition	7 (5)
Diverticulitis	5 (3.5)
Ileus	3 (2)
Pancreatitis	3 (2)
Crohn's disease	3 (2)
Gastrointestinal fistula	3 (2)
Ischemic bowel	1 (1)

The surgical indications formed 58.5% (83 patients) of TPN indications, while the rest of the indications were medical reasons 41.5% (59 patients).

*n*, number of patients; TPN, total parenteral nutrition.

The duration of TPN administration ranged from 1 to 35 days with a mean ± SD duration of 10 ± 7.2 days. More than one-third of the patients received TPN for a period of 6–10 days. There was no significant difference in duration average between patients with medical indications and those with surgical indications (10.05 vs. 10.06 days respectively, *P* = 0.99).

The TPN treatment used in our sample included the following components: dextrose ranging 119–370 g (mean ± SD was 268 ± 51), protein ranging 30–155 g (mean ± SD was 92 ± 19), and lipids ranging 0–345 mL (mean ± SD was 235 ± 61).

### Hyperglycemia prevalence

After studying the final sample of 142 patients, we found out that 42 patients (29.6%) developed hyperglycemia after TPN initiation. More than half of the patients (52%) with TPN-induced hyperglycemia started developing elevated blood glucose in the first 3 days following TPN initiation. We looked at the highest blood glucose numbers recorded for each of the 42 patients and found out that blood glucose values exceeded 250 mg/dL in 10 patients (24%) after receiving TPN. Furthermore, we compared the average of blood glucose numbers collected during the 3 days before TPN administration to the blood glucose average in the 3 days post TPN in the hyperglycemia group. We noticed that the baseline glucose average increased by 18.5% in the first 3 days following TPN initiation.

### Characteristics of hyperglycemia group

We compared the patients with TPN-induced hyperglycemia (42 patients) with those who did not develop hyperglycemia post TPN (100 patients), as demonstrated in Table 2. There was no significant difference between these two groups in terms of average age, gender, or race.

**Table 2.** Patient Characteristics

Patient characteristics	All patients (*N* = 142)	Hyperglycemia (*n* = 42)	Nonhyperglycemia (*n* = 100)	P
Age (years), mean ± SD	69.5 ± 14	71.2 ± 13.5	68.8 ± 14.2	0.3421^a^
Male, *n* (%)	60 (42.3)	15 (35.7)	45 (45)	0.3072^b^
Race, *n* (%)				
White	131 (92.3)	36 (85.7)	95 (95)	0.0592^b^
African American	11 (7.7)	6 (14.3)	5 (5)	
BMI (kg/m^2^), mean ± SD	27.49 ± 8.41	29.43 ± 10.95	26.68 ± 6.98	0.0751^a^
BMI categories, *n* (%)				
Underweight	13 (9.2)	5 (11.9)	8 (8)	0.4923^c^
Normal weight	45 (31.7)	11 (26.2)	34 (34)	0.3453^c^
Overweight	32 (22.5)	5 (11.9)	27 (27)	**0.0243**^c^
Obesity	52 (36.6)	21 (50.0)	31 (31)	**0.0353**^c^
TPN duration (days), mean ± SD	10.06 ± 7.18	13.62 ± 8.73	8.56 ± 5.84	**<0.0011**^a^
TPN indications, *n* (%)				
Medical	59 (41.5)	12 (28.6)	47 (47)	**0.0422**^b^
Surgical	83 (58.5)	30 (71.4)	53 (53)	
History of previous TPN, *n* (%)				
Yes	15 (10.6)	6 (14.3)	9 (9)	0.3502^b^
No	127 (89.4)	36 (85.7)	91 (91)	
TPN components, mean ± SD				
Dextrose (g)	268 ± 51	272 ± 47	266 ± 52	0.4611^a^
Protein (g)	92 ± 19	93 ± 21	91 ± 19	0.5551^a^
Lipids (mL)	235 ± 61	234 ± 50	235 ± 65	0.9061^a^
Steroids use, *n* (%)				
Yes	8 (5.6)	4 (10)	4 (4)	0.1932^b^
No	134 (94.4)	38 (90)	96 (96)	
Location of admission, *n* (%)				
ICU	28 (19.7)	12 (28.6)	16 (16)	0.0862^b^
Non-ICU	114 (80.3)	30 (71.4)	84 (84)	
Comorbidities, *n* (%)				
Hepatic disease	7 (5)	1 (2)	6 (6)	0.3096^b^
Renal disease	24 (17)	7 (17)	17 (17)	1^b^
Pancreatic disease	11 (8)	3 (7)	8 (8)	0.8384^b^

*Bold* numbers indicate significant differences at *p* < 0.05.

Duration of TPN, surgical indications, and obesity were significantly higher in hyperglycemia group compared to nonhyperglycemia group.

^a^ Based on two sample *t*-test.

^b^ Based on chi-square test.

^c^ Based on two-prop *z*-test.

BMI, body mass index; ICU, intensive care unit; SD, standard deviation.

The duration of TPN use was significantly higher in the hyperglycemia group than in the nonhyperglycemia group (13.6 ± 8.7 days vs. 8.6 ± 5.9 days respectively, *P* < 0.001). We noticed that the percentage of hyperglycemia among patients who received TPN for more than 2 weeks was significantly greater than those who received TPN for <2 weeks (56% vs. 23.5% respectively, *P* < 0.001).

Hyperglycemia patients who were prescribed TPN for surgical indications were significantly more than the ratio of surgical patients in the nonhyperglycemia group (71.4% vs. 53% respectively, *P* = 0.042).

Although the average of BMI in the hyperglycemia group was slightly higher than it was in the nonhyperglycemia group, the difference was not quite statistically significant (29.4 vs. 26.7 kg/m^2^ respectively, *P* = 0.075). However, when we looked separately at the BMI subgroups, we found that there was a significant difference between the obesity prevalence among hyperglycemia patients when compared with nonhyperglycemia ones (50% vs. 31% respectively, *P* = 0.035). Surprisingly, we did not notice such a pattern when we looked at the overweight category, where the percentage of overweight patients in hyperglycemia group was significantly less than the one in the nonhyperglycemia group (11.9% vs. 27% respectively, *P* = 0.024).

Although the percentages of ICU admissions and concurrent steroids use were higher in hyperglycemia group compared to nonhyperglycemia one, they both failed to show a statistically significant difference between these two groups. Also, no statistical difference was noted in TPN components, history of previous TPN and associated comorbidities when comparing the two study groups.

### Predictors of TPN-induced hyperglycemia

In an effort to predict hyperglycemia after TPN administration, variables that might contribute were examined by applying a multivariable logistic regression model. Holding other variables at a fixed value, there is sufficient evidence to conclude that there is a 4.3% increase in the odds of getting TPN-induced hyperglycemia per every 1 year increase in age (odds ratio [OR] = 1.043, 95% confidence interval [CI]: 1.005–1.083, *P* = 0.028). In addition, for each day increase in duration of TPN, the odds of developing hyperglycemia increase by 10% (OR = 1.103, 95% CI: 1.037–1.174, *P* = 0.002).

We also found that the odds of getting hyperglycemia for steroids takers who receive TPN are 585% higher than the odds among nonsteroids takers (OR = 6.85, 95% CI: 1.09–43.5, *P* = 0.04). The odds of having hyperglycemia in patients with surgical indications for TPN are 212% greater than the odds among patients with medical indications (OR = 3.12, 95% CI: 1.215–7.937, *P* = 0.018). Moreover, when we compared obesity subgroup to overweight subgroup, we found out that the odds of experiencing hyperglycemia post TPN therapy in obese patients are 260% higher than the odds in overweight patients (OR = 3.6, 95% CI: 1.04–12.5, *P* = 0.044).

## Discussion

### Prevalence of TPN-induced hyperglycemia

In our retrospective study, we found out that hyperglycemia developed in almost one-third (29.6%) of the patients who received TPN treatment.

Looking at previous research (Table 3), we found that our result is in accord with a retrospective study conducted by Dodds et al.^9^ on 2747 patients who received TPN over a 7-year period. Researchers of that study defined hyperglycemia as any blood glucose value higher than 200 mg/dL and reported the incidence of hyperglycemia to be 27.7%. This result is close to the one we achieved in our study, taking into consideration the slight difference in hyperglycemia cutoff.

**Table 3.** Comparing Our Study with Previous Relative Studies in Literature

Study	Study design	n	Type of patients	Hyperglycemia cutoff	Hyperglycemia prevalence (%)
Lee et al.^11^	Retrospective	88	ICU patients	Mean blood glucose ≥140 mg/dL	67
Pleva et al.^8^	Retrospective	50	non-ICU patients	>200 mg/dL	44
**Our study**	**Retrospective**	**142**	**All patients**	**>180 mg/dL**	**29.6**
Dodds et al.^9^	Retrospective	2747	All patients	>200 mg/dL	27.7
Llop et al.^7^	Prospective	119	non-ICU patients	>180 mg/dL	21
Sarkisian et al.^10^	Retrospective	100	non-ICU patients	Mean blood glucose ≥180 mg/dL	17

Studies are ordered based on the hyperglycemia prevalence from the highest to the lowest. This study, in *bold* text, is comparable to other studies that included all patients.

Some studies reported a lower incidence of hyperglycemia in comparison to our study. Llop et al.^7^ performed a multicentric prospective observational study on 119 noncritically ill patients who received TPN treatment. Hyperglycemia (defined as blood glucose >180 mg/dL) was detected in 25 patients (21%). Their result is slightly less than the finding of our study, which might be related to the fact that they only studied noncritically ill patients and did not include ICU patients.

Additionally, Sarkisian et al.^10^ conducted a retrospective chart review on 100 non-ICU adult TPN patients and showed that 17 patients (17%) experienced hyperglycemia (defined as a mean blood glucose level of 180 mg/dL or greater). This rather low incidence of hyperglycemia could be attributed to multiple factors in their study, such as: using the mean value of blood glucose readings recorded in the first 9 days of TPN treatment (instead of using a single blood glucose value as an indicator of hyperglycemia), only including patients who received TPN for 7 days or higher, and excluding ICU and hemodialysis patients.

On the other hand, few studies reported a higher incidence of hyperglycemia than what we found in our research. Lee et al.^11^ performed a retrospective study on 88 nondiabetic critically ill patients and showed a significantly higher rate of hyperglycemia (67%). This might be related to adopting a lower cutoff for hyperglycemia (patients with daily mean blood glucose level ≥140 mg/dL were considered as the hyperglycemia group) and only counting ICU patients in their study.

Another retrospective study was performed by Pleva et al.^8^ on 50 non-ICU TPN patients and revealed that 22 patients (44%) experienced hyperglycemia (defined as at least one blood glucose value >200 mg/dL) while receiving TPN therapy. Although this study excluded critically ill patients and used a higher cutoff for hyperglycemia, they still achieved a higher incidence of hyperglycemia than our study. A possible explanation for such finding might be due to using a small sample size, which may alter their outcomes.

### Potential risk factors for TPN-induced hyperglycemia

#### TPN duration

In our study, duration of TPN therapy was found to be an important risk factor and an independent predictor for developing hyperglycemia in TPN patients. The percentage of hyperglycemia in our TPN sample more than doubled (23.5%–56%) when we compared patients with a duration <2 weeks versus those with a duration more than 2 weeks. Moreover, the risk of hyperglycemia was predicted to increase by 10% with each additional day in TPN duration.

This result is in accord with many previous studies indicating that duration of TPN treatment was correlated positively with blood glucose levels among patients on TPN.^6,16,21^ However, this outcome is contrary to that of Lee et al.^11^ and Olveira et al.^22^ who did not find a significant correlation between hyperglycemia and duration of TPN.

One of the possible explanations for this result is that the patients who require longer duration of TPN are more likely to be critically ill, which in turn results in longer hospital stay and higher risk for inpatient complications. This result might also be simply explained by presuming that the chances of reporting a single glucose value >180 mg/dL would be higher when patients stay on TPN therapy for a prolonged period of time.

#### Surgical indications

Another significant risk factor for hyperglycemia reported in our study is having a surgical etiology as an indication for TPN therapy. Our study showed that the odds of getting hyperglycemia in surgical TPN patients are three times greater than those with medical reasons. This risk factor was also reported by Llop et al.^7^ when they linked hyperglycemia complication to performing surgical procedures within the 7 days before TPN initiation. This can be possibly explained by the degree of stress associated with surgical interventions.

#### Obesity

Our study found a significant difference in the obesity prevalence between hyperglycemia and nonhyperglycemia groups. Half of TPN patients who developed hyperglycemia in our sample were obese. Although our study failed to show that BMI as a continuous variable has a significant impact on developing hyperglycemia, it did show that the obesity subgroup is a significant risk factor for TPN-induced hyperglycemia.

Literature review revealed conflicting results regarding the effect of BMI on blood glucose levels among TPN patients. Olveira et al.^22^ found that higher BMI was significantly associated with hyperglycemia, whereas Llop et al.^7^ did not find a remarkable impact of BMI on serum blood glucose. Swainson et al.^23^ have shown that waist-height ratio is a better indicator than BMI in determining obesity. It was not possible to assess this indicator in our subjects due to the unavailability of waist circumference in the medical records. However, it is imperative in future studies to investigate whether this ratio has a more consistent relationship to TPN-induced hyperglycemia than BMI.

The relation between obesity and hyperglycemia risk can be attributed to the fact that obese patients have higher visceral fat deposition in their bodies, which is associated with multiple complications including metabolic syndrome and insulin resistance.^24^ Besides that, obese patients experience physiologic changes that may weaken their ability to accommodate the increased stress during critical illness.^25^

Surprisingly, our study revealed an inverse effect of overweight on blood glucose level compared to obesity. While the obesity prevalence was significantly higher in hyperglycemia group, the percentage of overweight patients in this group was remarkably lower when compared to nonhyperglycemia group. Additionally, the odds of developing hyperglycemia among obese patients were three times greater than the odds in overweight patients. We could not find in the literature a study similar to ours that examined this particular point. Interestingly, such discrepancy between overweight and obesity has been reported in different kind of studies that assessed the effect of BMI on mortality among cancer patients. Both Kroenke et al.^26^ and Caan et al.^27^ found out that overweight was associated with decreased mortality rates in contrary to obesity in cancer patients. Overweight patients have both sufficient muscle mass and modest adiposity, which make them at better nutritional status without falling into the obesity category where excess adipose tissue may cause insulin resistance and metabolic disorders.^27^

#### Other predictors

Corticosteroids are known to have hyperglycemia as a side effect of their administration. Steroids takers in our study had six times higher odds of developing hyperglycemia than nonsteroid takers. Pleva et al. had also reported such a link between hyperglycemia and steroids intake among TPN patients.^8^

In addition, age was found to have a considerable impact on hyperglycemia prediction in TPN patients (4% increase with each additional year in age). Similar finding had been reported by Edakkanambeth Varayil et al.^20^ where it was attributed to impaired insulin secretions along with declined serum insulin response to TPN therapy with increased age.

### Study limitations

The main limitation is the retrospective nature of our study. This retrospective method depends to a high extent on medical documentations that might not be sufficient in all cases. Additional hyperglycemia-related parameters could not be studied as we are limited to the information, diagnoses, and measurements that were collected at the time of care. Another limitation we faced during this study is that the diagnosis of diabetes mellitus was not consistently reported in all past medical histories of the patients. Thus, we stretched our exclusion criteria to not only reported diabetic patients, but also to any patient with an elevated blood glucose value before TPN. Other limitations to our study include being in a single center with the predominance of white race over other races.

## Conclusions

Our study showed that hyperglycemia is a common side effect of TPN therapy. The ability to anticipate the incidence of hyperglycemia among TPN patients is critical to minimize such complication and to optimize medical management for these patients. Thus, more attention should be paid to identify and recognize the risk factors of TPN-induced hyperglycemia to ensure a better glycemic control in such patients.
